# LncRNA NONHSAT114552 Sponges miR-320d to Promote Proliferation and Invasion of Chordoma Through Upregulating NRP1

**DOI:** 10.3389/fphar.2021.773918

**Published:** 2021-10-13

**Authors:** Kai Zhang, Zixiang Liu, Yingchuang Tang, Xiaofeng Shao, Xi Hua, Hao Liu, Huilin Yang, Kangwu Chen

**Affiliations:** Department of Orthopedic Surgery, The First Affiliated Hospital of Soochow University, Suzhou, China

**Keywords:** NONHSAT114552, miR-320d, NRP1, proliferation, invasion, chordoma

## Abstract

Chordoma is a relatively rare malignant bone tumor with high local recurrence. To date, the mechanism remains unclear. lncRNAs play a pivotal role in tumorigenesis by acting as competitive endogenous RNAs of microRNAs. However, the biological role of lncRNA is still unclear in chordoma. In this research, our aim is to investigate the roles and regulation mechanisms of lncRNA NONHSAT114552 in chordoma development. The expression level of NONHSAT114552 and miR-320d in chordoma tissues was determined by qRT-PCR. Meantime, the correlation between NONHSAT114552 and clinical prognosis was also studied. Bioinformatics analysis and luciferase reporter assays were used to verify the relationship between NONHSAT114552 and miR-320d, and between miR-320d and Neuropilin 1 (NRP1). In addition, effects of NONHSAT114552 on chordoma cells (U-CH1 and U-CH2) proliferation and invasion and its regulation on miR-320d were also evaluated. Furthermore, the influences of NONHSAT114552/miR-320d/NRP1 axis on chordoma tumorigenesis were investigated *in vivo*. NONHSAT114552 was overexpressed while miR-320d was down-regulated in chordoma tissue compared to fetal nucleus pulposus. Kaplan-Meier survival analysis showed that NONHSAT114552 overexpression was associated with patients’ poor prognosis. Knockdown of NONHSAT114552 significantly suppressed chordoma cell proliferation and invasion. *In vitro* studies confirmed that NONHSAT114552 acted as ceRNA to regulate NRP1 by directly sponging miR-320d, thus facilitating chordoma cell proliferation and invasion. *In vivo* study demonstrated that NONHSAT114552 moderated chordoma growth by sponging miR-320d to regulating NRP1. Our findings indicate that lncRNA NONHSAT114552 exhibits a critical role in the tumorigenesis and development of chordoma and it may become one potential prognostic marker and therapeutic target for this disease. .

## Introduction

Chordoma is a rarely primary malignant bone tumor, which is believed to originate from embryonic residual notochord tissue (Denaro et al., 2020). According to previous reports, its annual incidence is about 0.08/100,000 and it tends to occur in the sacrum of adults (Kayani et al., 2014; Zou et al., 2015a). Chordoma exhibits resistance to conventional chemoradiotherapy, and complete surgical excision is the most effective method for patients to achieve disease-free survival (George et al., 2015). However, many patients suffer local recurrence and metastasis even if they have received complete tumor resection. It is reported that local recurrence rate is as high as 30–85%, and the postoperative local recurrence rate is considered to be the most important predictor of death (Chen et al., 2011; Kayani et al., 2014; Ruosi et al., 2015; Denaro et al., 2020). Through the preliminary clinical studies, we have found that local recurrence of chordoma is partly related to the invasive nature of the tumor itself (Chen et al., 2011; Zhang et al., 2014a). Although there are many studies on the mechanism of chordoma recurrence and important progress has been made, the pathogenesis of chordoma is still unclear, ideal and effective therapeutic target has not been found yet.

Long noncoding RNA (lncRNA) is a category of noncoding RNA (ncRNA) whose transcriptional length exceeds 200 nucleotides (Flynn and Chang, 2014). A great deal of research has indicated that lncRNA exhibits vital roles in regulating tumor cell proliferation, invasion and migration (Chen et al., 2017a; Slaby et al., 2017). For example, lncRNA NONHSAT113026 overexpression can restrain the migration and invasion of renal cell cancer (Pu et al., 2019). More and more evidence has manifested that lncRNA can function as a diagnostic and prognostic factor for various tumors such as breast cancer (Zhou et al., 2019), lung cancer (Li et al., 2020a)and hepatocellular carcinoma (Lu et al., 2020). Whereas, little is known about the molecular regulation mechanisms of lncRNA in chordoma development.

MicroRNA (miRNA) is a kind of small non-coding RNAs which has a length of 18–25 nucleotides. Accumulating evidence has indicated that miRNA has great roles in affecting the occurrence and development of chordoma (Zhang et al., 2018; Yao and Wu, 2019). MiRNA-1 can inhibit chordoma invasion and migration by targeting the expression of Slug gene (Osaka et al., 2014). MiRNA-34a and miRNA-608 affect the cell invasion of chordoma by suppressing MET and EGFR (Zhang et al., 2014b). In recent years, cross-regulation between microRNAs and lncRNAs has captured wide attention. For instance, lncRNA UICLM can promote colorectal cancer metastasis by sponging miRNA-215 to affect ZEB2 expression (Chen et al., 2017b). In breast cancer, lncRNA LINC00899 was reported to suppress tumor progression by restraining miR-425 (Zhou et al., 2019).

Accumulating evidence has shown that miR-320d plays a critical role in the progression of numerous cancers and it’s down-regulation is closely associated with patients’ poor prognosis (Qin et al., 2017; Liu et al., 2019). Consistently, our previous research has also uncovered that miR-320d is down-regulated in chordoma (Chen et al., 2017c), but the detailed molecular mechanism remains unclear. Based on chip screening and bioinformatics analysis, we find that lncRNA NONHSAT 114552 may function as a potential ceRNA for miR-320d and play a role in chordoma development. Hence, more information about functional role of lncRNA NONHSAT114552 and its association with miR-320d in chordoma needs to be explored in chordoma.

## Materials and Methods

### Patients and Clinical Tissue Specimens

In current study, 25 chordoma patients who received primary tumor resection from 2009 to 2019 at the First Affiliated Hospital of Soochow University (Suzhou, China) were enrolled. None of the enrolled patients received preoperative anti-tumor treatment. All the patients took MR imaging regularly and continuous disease-free survival time (CDFS) was defined as the time interval from primary tumor resection to first recurrence. As control group, fetal nucleus pulposus (FNP) samples were obtained from 10 aborted fetuses with a gestational age of 12–28 weeks in the Department of Gynecology and Obstetrics. This research was approved by the Ethics Committee of First Affiliated Hospital of Soochow University. All the specimens were collected and stored in liquid nitrogen.

### Cell Culture and Transfection

Two chordoma cell lines, including U-CH1 and U-CH2, were purchased from American Type Culture Collection (ATCC, United States). These 2 cell lines were cultured in IMDM-RPMI media (4:1) supplemented with 10% fetal bovine serum and 1% penicillin-streptomycin. The transfection was performed by using a Lipofectamine 3,000 according to manufacturer’s instructions (Invitrogen, United States). Transfection plasmids (scramble, sh-NONHSAT114552, Lv-NRP1, Lv-NC, miR-NC, miR-320d inhibitor, NONHSAT114552-WT, NONHSAT114552-Mut) were obtained from GenePharma (China).

### RNA Isolation and Quantitative Real-Time PCR (qRT-PCR)

The total RNA was extracted from the tissues (chordoma and FNP) and cell lines (U-CH1 and U-CH2) using TRIzol reagent according to the manufacturer’s recommendations (Beyotime, China). Subsequently, the extracted RNA was reverse-transcribed to cDNA using a PrimeScript RT Reagent Kit (TaKaRa, Japan). SYBRGreen (TaKaRa, Japan) was used to perform real-time PCR. Relative expression levels of NONHSAT114552 and NRP1 were quantified based on the expression levels of GAPDH while the relative expression level of miR-320d was quantified depending on expression level of U6. The relative expression levels were calculated using 2 ^−ΔΔCT^ method. The primers used were shown as follows:

**Table:** 

Genes	Forward primer (5’-3’)	Reverse primer (5’-3’)
NONHSAT114552	CTT​GGC​CAC​TGG​GCA​CAG​AA	CTC​CCA​CCT​CAC​CAG​GAC​CA
miR-320d	AAA​AGC​TGG​GTT​GAG​AGG​A	TGGTGTCGTGGAGTCG
U6	GCT​TCG​GCA​GCA​CAT​ATA​CTA​AAA​T	CGC​TTC​ACG​AAT​TTG​CGT​GTC​AT
NRP1	GCG​CTA​CCA​GAA​GCC​AGA​GG	CTG​GCG​TGC​TCC​CTG​TTT​CA
GAPDH	GCG​GGG​CTC​TCC​AGA​ACA​TC	TCC​ACC​ACT​GAC​ACG​TTG​GC

### Western Blot Analysis

The total proteins were extracted from U-CH1 and U-CH2 cell lines using protein lysates (Beyotime, China). The proteins were separated by 10% sodium dodecyl sulphate-polyacrylamide gel electrophoresis and transferred onto a nitrocellulose membrane. After blocking with BSA, the membranes were incubated with primary antibodies against NRP1 (Abcam, United States) and internal reference GAPDH (Beyotime, China). Then, the bands were treated with secondary antibody and were developed using chemiluminescence substance (Beyotime, China).

### Fluorescence *in Situ* Hybridization (FISH) Assay

NONHSAT114552 probes were designed and synthesized by RiboBio (China). The probe signals were detected with a FISH Kit (RiboBio, China) according to the manufacturer’s recommendations. U-CH1 cells were fixed in 4% formalin and were hybridized at 37°C for 30 min in hybridization solution after prehybridization in PBS. The cell nuclei underwent counterstaining by using DAPI staining (Beyotime, China). At last, fluorescence microscope was used to capture the images.

### Cell Proliferation, Invasion and Migration Assays

Cell proliferation was determined by Cell Counting Kit-8 (CCK8) (Dojindo, Japan) and colony formation assays. For CCK8 assay, U-CH1 or U-CH2 cells were inoculated to 96-well plates. Cell culture was terminated at 24, 48, 72 h respectively. Optical density (OD) value at 450 nm was examined. For colony formation assay, U-CH1 or U-CH2 cells with different transfection were seeded into 6-wells plates (5×10^3^ cells/well) for 2 weeks. Then, the colonies were fixed with methanol and stained with 1% crystal violet. Next, the number of colonies was observed via a microscope and Image software. Cell invasion was evaluated by transwell chambers (8.0 μm, Corning, United States). After incubation for 24 h, the invaded cells were fixed with paraformaldehyde and stained with crystal violet. Five fields were randomly selected for counting in each chamber under an inverted microscope. In wound healing assay, the transfected cells (1×10^6^) were seeded in a 6-well plate and cultured to approximately 80% confluence. Subsequently, we scraped cells with 200 μL plastic head to form a straight wound. The 0 and 24 h wound area data were recorded under a microscope. All the tests were done thrice.

### Dual Luciferase Reporter Assay

The recombinant plasmid pisCHECK-2-NONHSAT114552-WT/Mut or pisCHECK-2-NRP1-WT/Mut was constructed in by OE Biotech Co., Ltd (China). Cells were seeded in 24‐well plates and co-transfected with wild‐type/mutated NONHSAT114552 constructs and miR-320d mimic or wild‐type/mutated NRP1 constructs and miR-320d mimic. Fluorescence signal changes were assessed and normalized to firefly luciferase activity by using the dual-luciferase reporter assay system (Promega, United States).

### Bioinformatics Prediction and Analysis

Independent online tool miRanda was used for predicting the potential binding site of NONHSAT114552 targeting microRNA-320d. TargetScan v7.2, microRNAorg and PITA were applied to detect target gene NRP1 and miR-320d binding site of NRP1 and forecast the potential downstream target genes of NONHSAT114552-miR-320d.

### Tumor Xenograft Model and Tumor Formation Assay

In this study, 8-week-old male NOD/SCID mice were injected subcutaneously with U-CH1 cells (1×10^7^ cells/mouse, 0.2 ml) which were stably transfected with sh-NONHSAT114552 or Lv-NRP1+sh-NONHSAT114552. Chordoma tissue volumes were evaluated and counted every 5 days according to the formula: tumor volume = 1/2 × length × width × width. On day 25 post-injection, the subcutaneous xenograft tumors were resected and the tumor growth was examined.

### Statistical Analysis

All the data in this study was shown in as mean ± SD. All statistical analyses were performed by making use of SPSS 19.0 software or GraphPad Prism 8. The difference between two groups was assessed by Student’s t test and if *p* < 0.05, the difference was considered statistically significant (*p < 0.05, **p < 0.01, ***p < 0.001).

## Results

### lncRNA NONHSAT114552 Is Up-Regulated While miR-320d Is Down-Regulated in Chordoma

**FIGURE 1:**
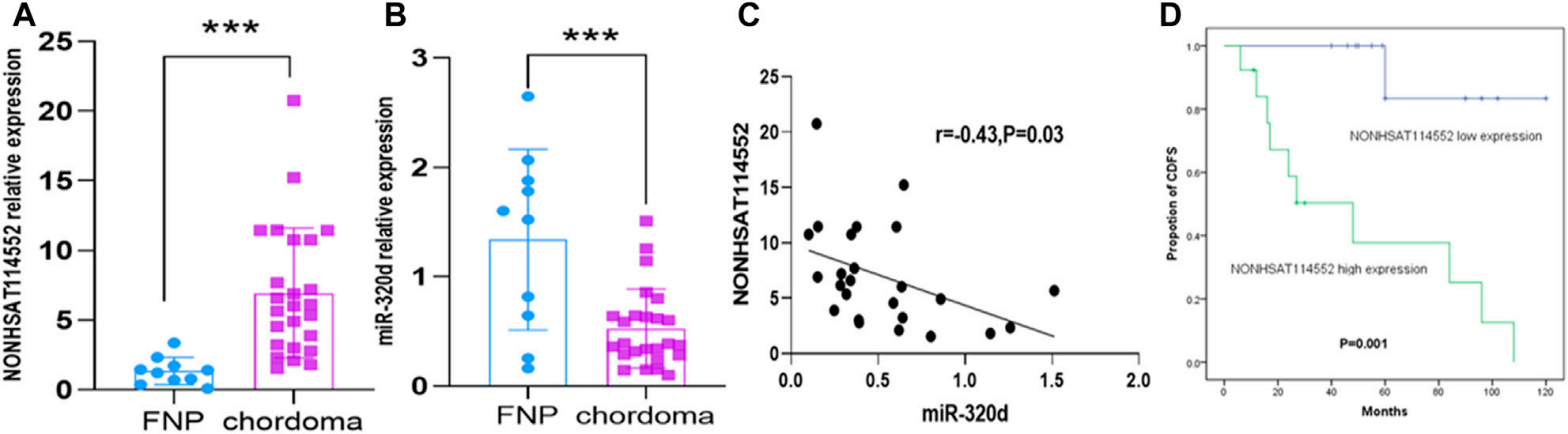
Overexpression of NONHSAT114552 in chordoma and its relation to patients’ prognosis **(A, B)** qRT-PCR showed that lncRNA NONHSAT114552 was overexpressed and miR-320d was reduced in chordoma **(C)**. Pearson correlation test showed negative relationship between NONHSAT114552 and miR-320d. **(D)**. CDFS on the basis of NONHSAT114552 expression in chordoma.

To study the effect of lncRNA NONHSAT114552 on chordoma progression, we first evaluated the NONHSAT114552 expression in 25 chordoma tissues and 10 fetal nucleus pulposus. Compared to FNP, qRT-PCR showed that NONHSAT114552 was significantly up-regulated while miR-320d was down-regulated in chordoma tissues (Figures 1A,B). Pearson correlation test showed close relationship between NONHSAT114552 and miR-320d (r = -0.43, *p* = 0.03) (Figure 1C). In addition, we also explored the potential of NONHSAT114552 as a prognostic biomarker. Kaplan–Meier survival curve and log-rank test demonstrated that patients with NONHSAT114552 high expression had a shorter CDFS than those who with NONHSAT114552 low expression, indicating that NONHSAT114552 might have vital prognostic value for chordoma patients (Figure 1D).

### The Effect of NONHSAT114552 Expression on Chordoma Cell Proliferation, Invasion and Migration *In Vitro*

**FIGURE 2:**
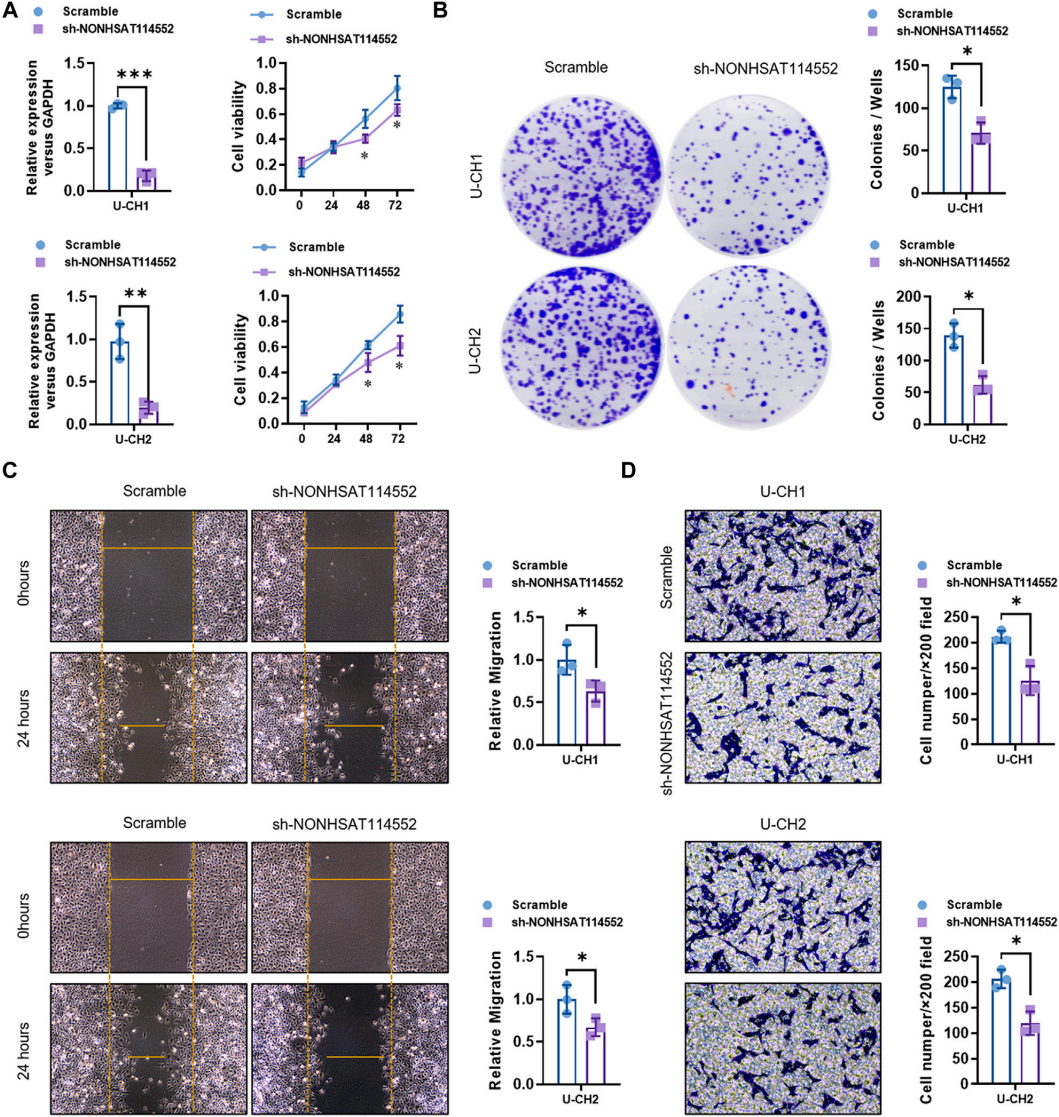
Knockdown of NONHSAT114552 affected chordoma cells (U-CH1 and U-CH2) proliferation, invasion and migration **(A)**. Left, NONHSAT114552 relative expression was assessed after U-CH1 or U-CH2 cells were transfected with sh-NONHSAT114552. Right, knockdown of NONHSAT114552 significantly inhibited U-CH1 or U-CH2 cell viability **(B)**. Colony formation assays showed that knockdown of NONHSAT114552 significantly restrained the proliferation of U-CH1 or U-CH2 cells **(C)**. Wound healing assays demonstrated that deletion of NONHSAT114552 refrained migration of chordoma cells **(D)**. Transwell assays were conducted for assessing U-CH1 or U-CH2 cell invasion with NONHSAT114552 knockdown.

To explore the function of NONHSAT114552 in chordoma, we knocked down NONHSAT114552 expression in chordoma cell lines (U-CH1 and U-CH2) by using sh-NONHSAT114552 (Figure 2A). qRT-PCR analysis verified the knockdown efficiency (Figure 2A). CCK8 and colony formation assays showed that U-CH1 and U-CH2 cell proliferation was both significantly inhibited after transfected with sh-NONHSAT114552 (Figures 2A,B). Transwell assay results demonstrated the invasion ability of U-CH1 or U-CH2 cells was dramatically suppressed with NONHSAT114552 silencing (Figure 2D). Similarly, wound healing assay exhibited the same conclusion that NONHSAT114552 deletion could inhibit the migration of chordoma cells (Figure 2C).

### NONHSAT114552 Functions as a ceRNA Directly Binding to miR-320d in Chordoma

**FIGURE 3:**
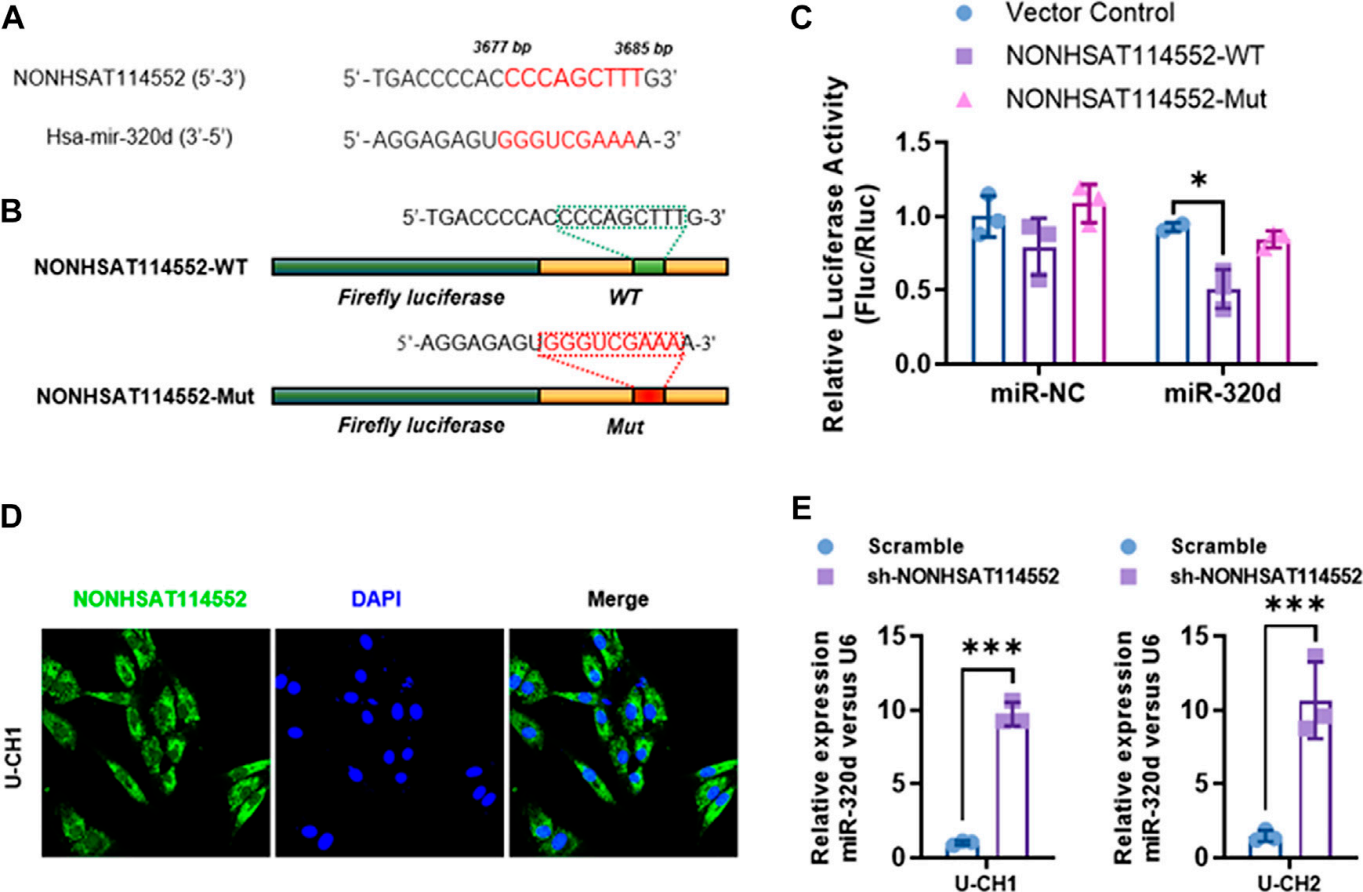
NONHSAT114552 directly targets miR-320d in chordoma **(A, B)**. Bioinformatic analysis identified miR-320d binding sites in the 3′UTR of NONHSAT114552 and luciferase reporter plasmid including NONHSAT114552-WT or NONHSAT114552-Mut was subsequently shown **(C)**. On the basis of luciferase reporter assay, miR-320d significantly decreased luciferase signals of NONHSAT114552-WT reporters, but did not affect NONHSAT114552-Mut reporters **(D)**. FISH assay showed location of NONHSAT114552 in U-CH1 cells **(E)**. miR-320d relative expression level was significantly up-regulated after knockdown of NONHSAT114552.

Numerous studies have demonstrated that lncRNA can act as competing endogenous RNA (ceRNA) for microRNA in different cancers (Chen et al., 2017b; Li et al., 2020a; Lu et al., 2020). In chordoma tissue, we found that NONHSAT114552 expression negatively correlated with miR-320d (Figure 1C). Moreover, bioinformatics analysis predicted that NONHSAT114552 could be a molecular sponge of miR-320d. The two predicted binding sites of NONHSAT114552 and miR-320d were shown in Figure 3A. To validate the interaction between miR-320d and NONHSAT114552, luciferase reporter assay was performed containing a wild-type (WT) or mutated miR-320d binding site in NONHSAT114552 (Figure 3B). The results indicated that miR-320d significantly reduced the luciferase signals of NONHSAT114552-WT reporters, but exhibited no influence on NONHSAT114552-Mut reporters (Figure 3C). In addition, FISH assay indicated that the location of lncRNA-NONHSAT114552 was mainly in the cytoplasm of U-CH1 cells (Figure 3D). Furthermore, miR-320d expression was significantly up-regulated after U-CH1 or U-CH2 cells were transfected with sh-NONHSAT114552(Figure 3E). Taken together, these data indicate that NONHSAT114552 acts as a ceRNA sponging miR-320d in chordoma.

### miR-320d Inhibition Rescued the Effects of NONHSAT114552 Knock Down on Chordoma Cells Proliferation, Invasion and Migration *In Vitro*

**FIGURE 4:**
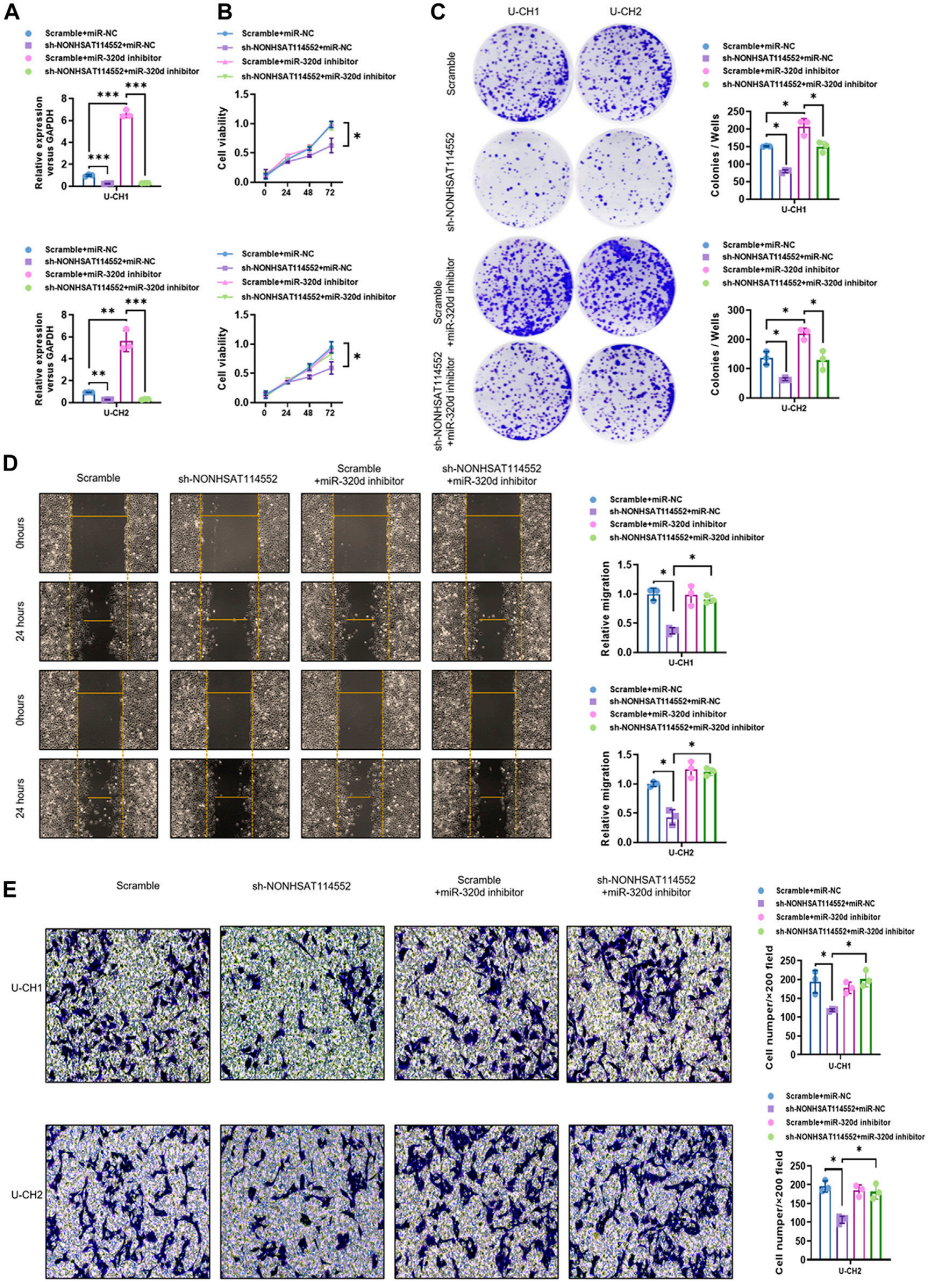
miR-320d suppression rescued the effects of NONHSAT114552 knockdown on chordoma cell proliferation, invasion and migration **(A)**. NONHSAT114552 mRNA expression was measured in different groups **(B)**. Cell viability of U-CH1 or U-CH2 cells in different groups was detected by CCK8 **(C)**. Colony of U-CH1 or U-CH2 cells in different groups was assessed by colony formation assays **(D)**. Migration ability of U-CH1 or U-CH2 cells in different groups was evaluated by wound healing assays **(E)**. Invasion ability of U-CH1 or U-CH2 cells in different groups was analyzed by Transwell assays.

To determine whether miR-320d refers to mediating the effect of NONHSAT114552 on chordoma cells, rescue experiments were constructed. qPCR analysis confirmed the transfection efficiency of U-CH1 or U-CH2 cells (Figure 4A). CCK8 and colony formation assay results showed that the suppression of U-CH1 or U-CH2 cell proliferation mediated by NONHSAT114552 knockdown was rescued by co-transfection with miR-320d inhibitor (Figures 4B,C). In addition, transwell and wound healing assays were done to assess chordoma cell invasion and migration in different groups. Transwell assay results demonstrated that sh-NONHSAT114552-induced invasion of U-CH1 or U-CH2 cells was alleviated by miR-320d knockdown (Figure 4E). Meanwhile, the wound healing assay results indicated that sh-NONHSAT114552-induced migration of U-CH1 or U-CH2 cells was significantly relieved by miR-320d knockdown (Figure 4D). In short, influences of NONHSAT114552 on cell proliferation, invasion and migration were mediated by miR-320d.

### Neuropilin-1(NRP1) is a Target Gene of miR-320d and it is Regulated by NONHSAT114552 and miR-320d

**FIGURE 5:**
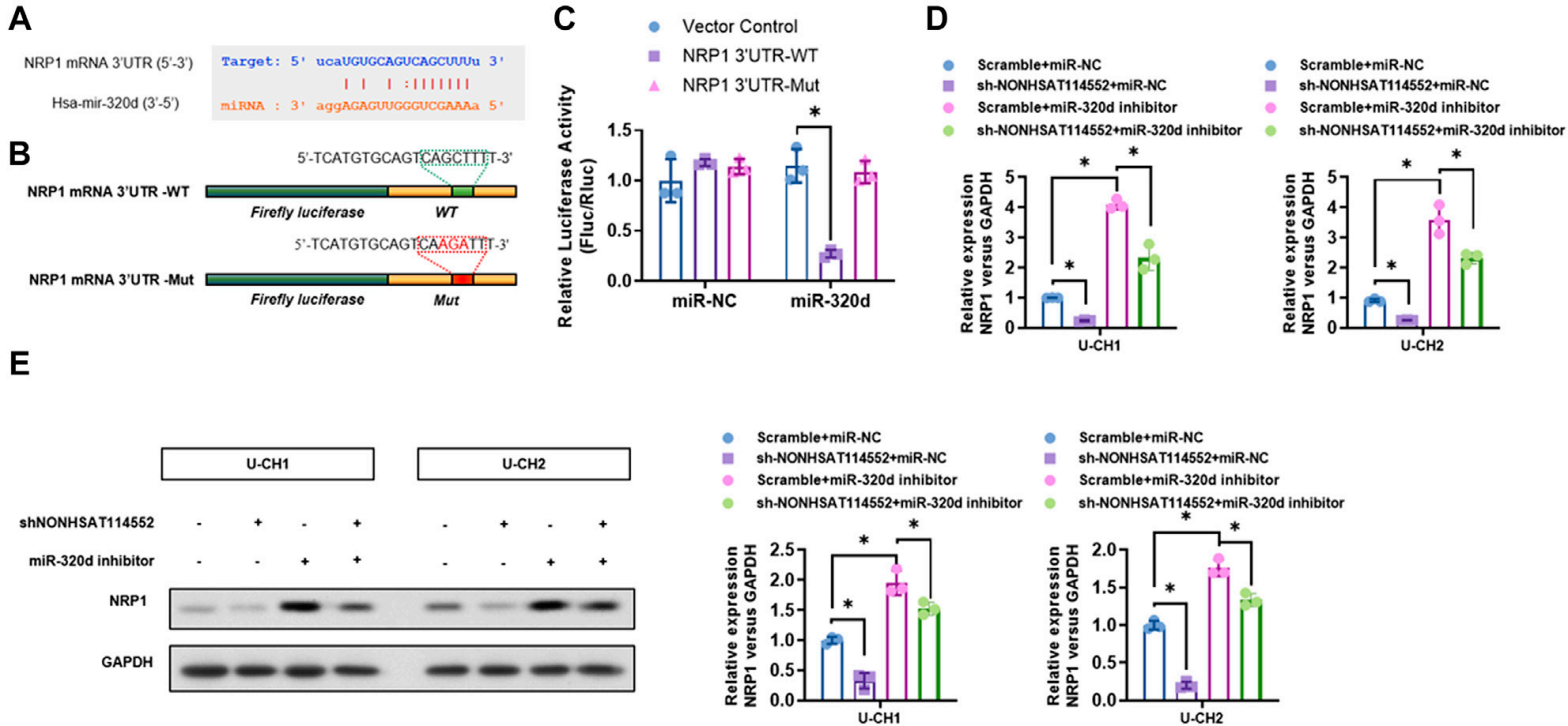
NRP1 is one target gene of miR-320d and regulated by NONHSAT114552 and miR-320d. **(A)**. Bioinformatic analysis predicted miR-320d target site in the 3′UTR of NRP1 **(B)**. The picture indicated luciferase reporter plasmid including WT or Mut NRP1 **(C)**. On the basis of luciferase reporter assay, miR-320d significantly decreased luciferase signals of NRP1-WT reporters, but did not affect NRP1-Mut reporters **(D)**. NRP1 mRNA expression was investigated in groups of sh-NONHSAT114552+miR-320d inhibitor, miR-320d inhibitor, sh-NONHSAT114552 or scramble **(E)**. NRP1 Protein expression was measured in groups of sh-NONHSAT114552+miR-320d inhibitor, miR-320d inhibitor, sh-NONHSAT114552 or scramble.

Bioinformatics analysis demonstrated that NRP1 may be one candidate target gene of miR-320d and 3′UTR of NRP1 may include potential binding site of miR-320d (Figure 5A). A great deal of research indicated that NRP1 can facilitate cell proliferation, invasion and migration in numerous tumor types (Lu et al., 2008; Bergé et al., 2011). In addition, NRP1, which functions as a co-receptor of VEGF and VEGF receptor, can also motivate tumor angiogenesis (Wu et al., 2014). In our study, WT 3′UTR sequence of NRP1 which includes miR-320d binding site (NRP1 3′UTR-WT), or mutant 3′UTR sequence of NRP1 containing predicted miR-320d binding site (NRP1 3′UTR-Mut) was used in luciferase reporter assays (Figure 5B). The results manifested that miR-320d overexpression significantly reduced luciferase activity with NRP1 3′UTR-WT, while this suppression was eliminated by the mutation of the miR-320d binding site in the NRP1 3′UTR (Figure 5C). Furthermore, whether miR-320d was involved in the relation between NONHSAT114552 and NRP1 was also investigated. Our data uncovered that miR-320d inhibitor significantly reversed the inhibiting effect of shNONHSAT114552 on NRP1 expression (Figures 5D,E). Therefore, NONHSAT114552 can affect the NRP1 mRNA and protein expression by modulating miR-320d.

### NRP1 Alleviated the Suppression of NONHSAT114552 on the Proliferation, Invasion and Migration of Chordoma Cells *In Vitro*

**FIGURE 6:**
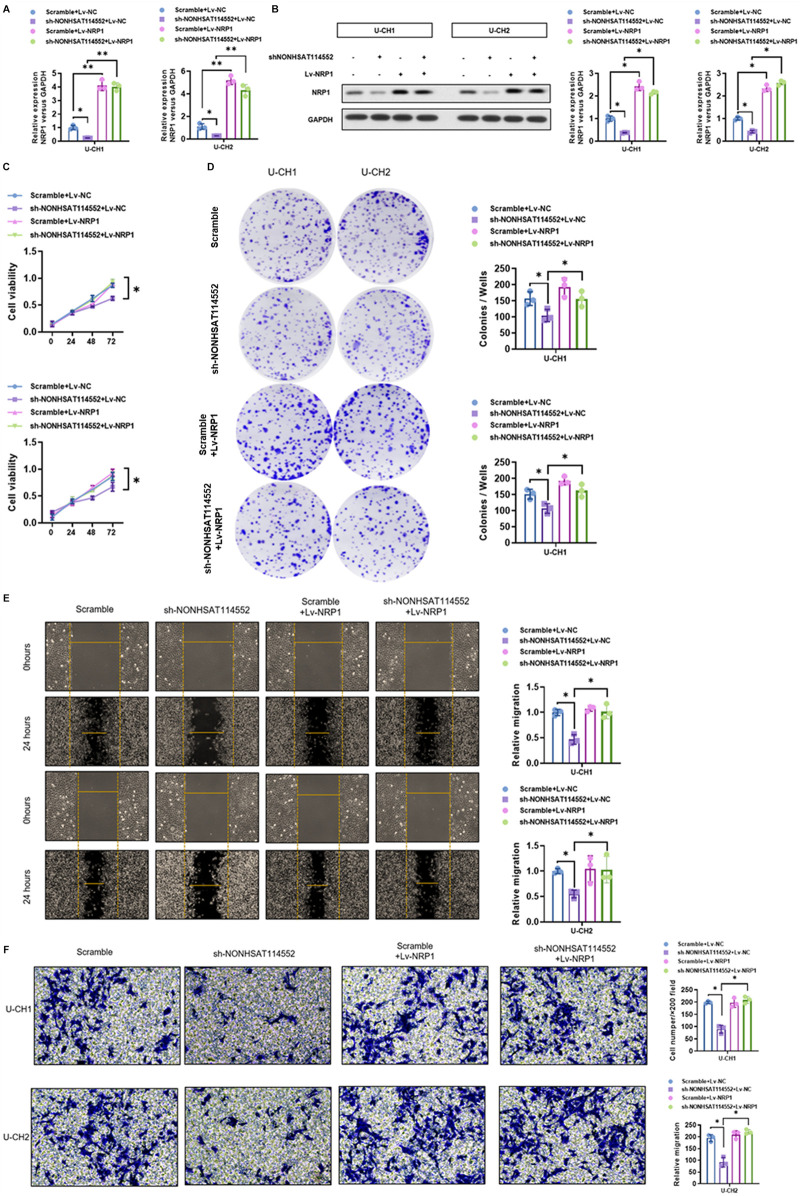
NRP1 alleviated the suppression of NONHSAT114552 on chordoma cell proliferation, invasion and migration **(A, B)**. NRP1 mRNA and protein expression level was assessed by qRT-PCR and WB in groups of sh-NONHSAT114552+Lv-NRP1, Lv-NRP1, sh-NONHSAT114552 or scramble **(C)**. Cell viability of U-CH1 or U-CH2 cells was detected by CCK8 assays in groups of sh-NONHSAT114552+Lv-NRP1, Lv-NRP1, sh-NONHSAT114552 or scramble **(D)**. Colony of U-CH1 or U-CH2 cells was measured by colony formation assays in groups of sh-NONHSAT114552+Lv-NRP1, Lv-NRP1, sh-NONHSAT114552 or scramble **(E)**. Migration ability of U-CH1 or U-CH2 cells in groups of sh-NONHSAT114552+Lv-NRP1, Lv-NRP1, sh-NONHSAT114552 or scramble was investigated by wound healing assays **(F)**. The cell invasive ability changes of U-CH1 or U-CH2 cells were analyzed by transwell assays in groups of sh-NONHSAT114552+Lv-NRP1, Lv-NRP1, sh-NONHSAT114552 or scramble.

The influences of NONHSAT114552 knockdown on the level of NRP1 mRNA and protein expression were investigated in U-CH1 or U-CH2 cells. qRT-PCR and Western blot (WB) analysis demonstrated co-transfection with sh-NONHSAT114552 and Lv-NRP1 can rescue NRP1 expression suppressed by transfection with sh-NONHSAT114552 alone (Figures 6A,B). CCK8 and colony formation assays manifested that suppression of U-CH1 or UCH-2 cell proliferation induced by NONHSAT114552 knockdown was alleviated when the cells were co-transfected with Lv-NRP1 (Figures 6C,D). Transwell assays manifested that NONHSAT114552 knockdown could significantly inhibit the chordoma cell invasion and such inhibition could be eliminated by re-expressing NRP1 (Figure 6F). Similarly, wound healing assays demonstrated the inhibition of cell migration produced by sh-NONHSAT114552 was reversed by NRP1 re-expression (Figure 6D).

### Effects of NONHSAT114552/miR-320d/NRP1 Axis on Tumorigenesis of Chordoma *In Vivo*

**FIGURE 7:**
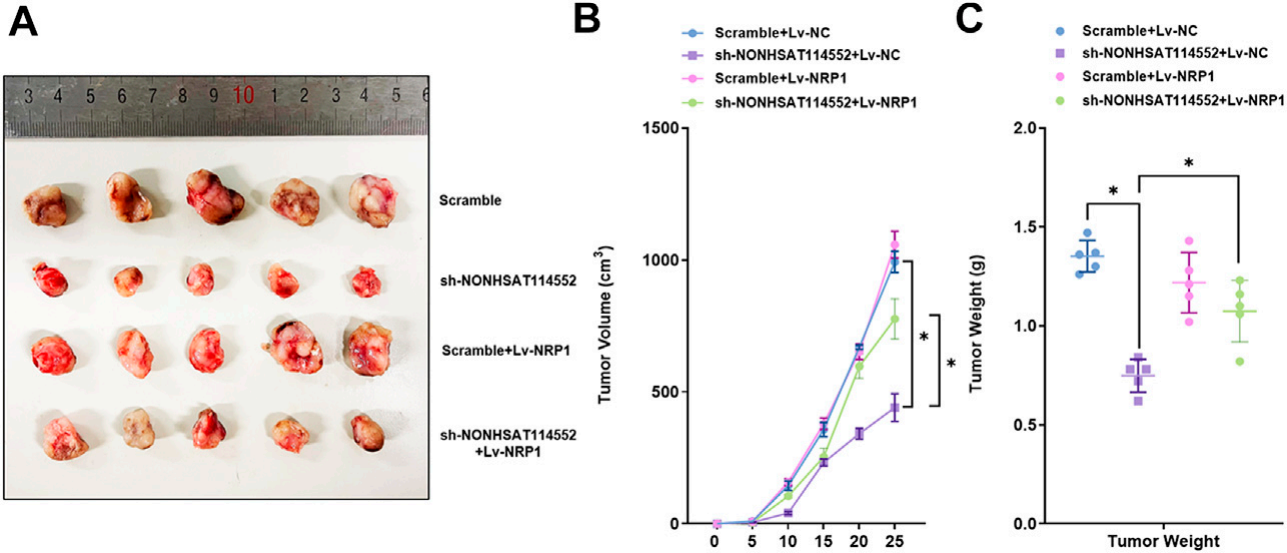
Effects of NONHSAT114552/miR-320d/NRP1 axis on tumorigenesis of chordoma in vivo **(A)**. Appearance of tumors formed in shNONHSAT114552, shNONHSAT114552+Lv-NRP1, Lv-NRP1 or control group respectively **(B)**. Tumor volume was assessed from each group at different point of time **(C)**. Tumor weight was evaluated in every group.

To verify effects of NONHSAT114552/miR-320d/NRP1 axis on chordoma tumorigenesis, U-CH1 cells which were co-transfected with sh-NONHSAT114552/Lv-NRP1 or control vector were subcutaneously injected into NOD/SCID mice respectively. As demonstrated in Figure 7A, deletion of NONHSAT114552 suppressed chordoma tumor growth *in vivo*. Similarly, tumor volume and weight were significantly reduced in sh-NONHSAT114552 group compared to that in control group, but this decrease was rescued in sh-NONHSAT114552+Lv-NRP1 group (Figures 7B,C). Taken together, NONHSAT114552 moderates chordoma development through regulating NRP1 *in vivo*.

## Discussion

ncRNA has been acknowledged to exhibit pivotal roles in the occurrence and progression of multiple tumors (Chen et al., 2017b; Wang et al., 2017). Dysregulation of lncRNAs or miRNAs is reported to be associated with cancer progression, suggesting ncRNAs might be potential targets for tumor therapy (Osaka et al., 2014; Slaby et al., 2017; Lu et al., 2020). Actually, numerous of miRNAs are verified to serve as diagnostic markers and therapeutic agents for chordoma, but whether lncRNA exerts a role in chordoma remains unclear (Osaka et al., 2014; Ma et al., 2017; Yao and Wu, 2019). In this research, we identified a novel lncRNA, NONHSAT114552, which promoted chordoma progression. lncRNA NONHSAT114552 was up-regulated in chordoma tissue and the overexpression of NONHSAT114552 was negatively related to the prognosis of chordoma patients. Knockdown of NONHSAT114552 restrained chordoma cell proliferation, invasion and migration. Molecular mechanism analysis demonstrated that NONHSAT114552 can promote the proliferation and invasion of chordoma cells by acting as a ceRNA for miR-320d to regulate NRP1 expression.

Recent studies reported that some lncRNAs functioned as tumor suppressors, oncogenes to regulate chordoma cell invasion (Ma et al., 2017; Wang et al., 2020). For instance, LINC00662 can facilitate chordoma development by activating RNF144B (Wang et al., 2020). Ma X et al. reported that lncRNA LOC554202 modulated chordoma cell invasion by affecting EZH2 expression (Ma et al., 2017). Whereas, they did not screen for differentially expressed ncRNA in chordomas. Based on lncRNA-miRNA-mRNA chip screening and bioinformatics analysis in our study, lncRNA NONHSAT114552 was selected for investigating the function of lncRNA in chordoma. Compared to fetal nucleus pulposus (FNP), chordoma tissue has overexpression of NONHSAT114552 and has low-expression of miR-320d. Patients with NONHSAT114552 overexpression had a shorter CDFS than those with NONHSAT114552 deletion. These findings showed that NONHSAT114552 might become one prognosis predictor for chordoma.

Moreover, invitro study indicated that NONHSAT114552 knockdown could suppress cell proliferation in chordoma. NONHSAT114552 down-regulation could significantly attenuate chordoma cell invasion and migration. Meantime, deletion of NONHSAT114552 could restrain tumor growth significantly compared to control group *in vivo*. As far as I am concerned, this is the first study to uncover that NONHSAT114552 exhibits an oncogenic role in chordoma tumorigenesis. As a type of ncRNAs, miRNAs are widely involved in chordoma behaviour (Zhang et al., 2014b; Osaka et al., 2014; Zou et al., 2015b). For example, miR-16–5p is reported to be down-regulated in chordoma and it can suppress chordoma cell proliferation, invasion and metastasis by targeting Smad3 (Zhang et al., 2018). miR-100–5p exerts a crucial role in chordoma development and its upregulation can restrain the growth of chordoma (Zhang et al., 2020). Similarly, we discovered the down-regulation of miR-320d in chordoma tissue compared to FNP. However, the detailed mechanism is needed to investigate.

miR-320 has been reported to be related to tumor cell proliferation and migration (Wu et al., 2014; Zhu et al., 2018). In glioma, miR-320 is down-regulated and it can inhibit glioma cell growth by suppressing Pre-B cell leukemia homeobox 3 (Pan et al., 2017). miR-320d was identified to be a tumor suppression miRNA and it can restrain cancer progression (Liu et al., 2019; Chen et al., 2020). However, whether miR-320d exerts a role in chordoma progression and the molecular mechanism is still unknown. Bioinformatics analysis showed that NRP1 was probably one target gene of miR-320d. Accumulating studies have demonstrated that NRP1 can act as multiple co-receptors and promote cell invasion and migration in various types of tumors (Yue et al., 2014; Huang et al., 2018). In osteosarcoma, knockdown of NRP1 can suppress cell invasion and angiogenesis (Yue et al., 2014). In our present study, NRP1 is verified as one target gene of miR-320d. miR-320d may influence the chordoma progression by directly downregulating the expression of NRP1.

Recently, the ceRNA hypothesis has attracted extensive attention which proposes a novel regulatory nekwork involving lncRNAs, miRNAs and mRNAs. This network was confirmed to play critical roles in cancer development (Lu et al., 2020). lncRNA can act as a ceRNA to interact with miRNA so as to regulate downstream target genes. Bioinformatics analysis demonstrated that NONHSAT114552 was a potential ceRNA for miR-320d. In Esophageal Squamous Cell Carcinoma, LncRNA NLIPMT can inhibit tumorigenesis by regulating miR-320/Survivin axis (Li et al., 2020b). lncRNA titin-antisense RNA1 is overexpressed in cholangiocarcinoma and it can sponge miR-320a to regulate neuropilin-1 expression (Zhu et al., 2020). In our study, luciferase reporter assay confirmed that lncRNA NONHSAT114552 was directly combined with miR-320d. Furthermore, NONHSAT114552 indirectly regulated NRP1 expression by regulating miR-320d. The rescue assays showed that effects of NONHSAT114552 silencing on chordoma cell biological behaviour were eliminated by miR-320d suppression or NRP1 re-expression. Moreover, *in-vivo* study demonstrated that NONHSAT114552 controlled chordoma growth by sponging miR-320d to regulate NRP1. Taken together, our results suggested that NONHSAT114552 promoted chordoma development through regulating miR-320d/NRP1 axis.

## Conclusion

In summary, our current study manifested the aberrant expression of NONHSAT114552 in chordoma tissue and patients with NONHSAT114552 overexpression had a poor prognosis. Furthermore, invitro and invivo studies indicated that NONHSAT114552 acted as a ceRNA for miR-320d to regulate NRP1 in chordoma tumorigenesis and progression. Our study suggested that NONHSAT114552 might become a biomarker for diagnosis and treatment of chordoma in future. Whereas, on account of rarity of chordoma, the number of chordoma tissue in this study is limited, more cases will be studied in the next study.

## Data Availability

The original contributions presented in the study are included in the article/Supplementary Materials, further inquiries can be directed to the corresponding authors.
